# Chitin Oligosaccharide (COS) Reduces Antibiotics Dose and Prevents Antibiotics-Caused Side Effects in Adolescent Idiopathic Scoliosis (AIS) Patients with Spinal Fusion Surgery

**DOI:** 10.3390/md15030070

**Published:** 2017-03-14

**Authors:** Yang Qu, Jinyu Xu, Haohan Zhou, Rongpeng Dong, Mingyang Kang, Jianwu Zhao

**Affiliations:** Department of Orthopedics, The Second Hospital of JiLin University, Changchun 130041, China; quyang_22d@163.com (Y.Q.); xujy0913@sina.com (J.X.); zhouhh1609@sina.com (H.Z.); drpeng1507@sina.com (R.D.); kmy_fge@126.com (M.K.)

**Keywords:** chitin oligosaccharide, antimicrobial prophylaxis, adolescent idiopathic scoliosis, spinal fusion

## Abstract

Antibiotics are always considered for surgical site infection (SSI) in adolescent idiopathic scoliosis (AIS) surgery. However, the use of antibiotics often causes the antibiotic resistance of pathogens and side effects. Thus, it is necessary to explore natural products as drug candidates. Chitin Oligosaccharide (COS) has anti-inflammation and anti-bacteria functions. The effects of COS on surgical infection in AIS surgery were investigated. A total of 312 AIS patients were evenly and randomly assigned into control group (CG, each patient took one-gram alternative Azithromycin/Erythromycin/Cloxacillin/Aztreonam/Ceftazidime or combined daily), experiment group (EG, each patient took 20 mg COS and half-dose antibiotics daily), and placebo group (PG, each patient took 20 mg placebo and half-dose antibiotics daily). The average follow-up was one month, and infection severity and side effects were analyzed. The effects of COS on isolated pathogens were analyzed. SSI rates were 2%, 3% and 8% for spine wounds and 1%, 2% and 7% for iliac wound in CG, EG and PG (*p* < 0.05), respectively. COS reduces the side effects caused by antibiotics (*p* < 0.05). COS improved biochemical indexes and reduced the levels of interleukin (IL)-6 and tumor necrosis factor (TNF) alpha. COS reduced the antibiotics dose and antibiotics-caused side effects in AIS patients with spinal fusion surgery by improving antioxidant and anti-inflammatory activities. COS should be developed as potential adjuvant for antibiotics therapies.

## 1. Introduction

In most surgeries, surgical infection caused by pathogens is a common problem, which increases patients’ morbidity and prolongs the duration of hospital stay [1]. The risk of post-operative surgical infection is still increasing, especially in the surgery for adolescent idiopathic scoliosis (AIS). Azithromycin is the normally used antibiotics for preventing surgical site infections (SSIs). Erythromycin combined with the other methods has been proved an effective and safe method in seroma therapy in general surgery and traumatology [2]. However, long-term utilization of low-dose erythromycin after surgery was not recommended for surgical patients [3]. There are many ways for inhibiting the risk and the progression of SSI. Earlier results suggested that cloxacillin is effective for AIS therapy in methicillin-sensitive staphylococcal infections [3]. Aztreonam is a monobactam antibiotic mainly used to fight against an infection caused by Gram-negative aerobic bacteria. Aztreonam has been proven effective for prophylaxis and therapy of urinary tract infections after prostate surgery [4]. Antibiotic prophylaxis with ceftazidime has been proven effective for preventing surgical infections [5].

Although these antibiotics have been proved to be effective for preventing surgical infection after surgery, the trouble it that drug resistance is the main threat for public health with the widespread use of antibiotics and it is a big challenge to control drug-resistant pathogens [3,6]. The resistance of enterobacteriaceae to antibiotics has been steadily increasing [7]. The resistance of pathogens to cloxacillin [8,9], aztreonam [10,11] and ceftazidime [12,13] has been widely reported. The problem is becoming more pronounced because drug resistance accumulates faster than new antibiotics have been developed.

Furthermore, some adverse effects of these antibiotics limit their application. Side effects have been reported in azithromycin-treated patients (gastric upset, nausea, and headache) and there are some manifestations like vomiting or diarrhea and abdominal pain symptoms [14]. The application of cloxacillin will result in the side effects including hypotension, tachycardia, flushing, palpitation, headache and nausea [15]. cloxacillin induces seizure in a hemodialysis patient [16] and often causes fever, chills, malaise, abdominal pain and anxiety [17]. The most frequent side effects of azithromycin are associated with a gastrointestinal system, such as abdominal pain, diarrhea, and nausea [14]. The most frequent side effects for aztreonam are acute renal failure, skin rash, and eosinophilia [18]. Long-term use of ceftazidime will result in side effects such as fever, rash, and pancytopenia. Gastrointestinal side effects limit erythromycin treatment and compliance [19]. Therefore, it is critical to find a novel way to control drug-resistant bacteria and related infections [20], and it is necessary to explore natural products for antibiotic therapy.

Chitin, a long-chain polymer of an *N*-acetylglucosamine, is the most abundant natural resource on the earth. Chitin is the major component of crustacean (crab [21], lobster [22] and shrimp [23]) shells, the cell walls of fungi [24] and insects [25], and the radulae of Mollusca [26]. The safety of chitin has been proven as a carrier for drug delivery [27]. Chitin can be digested and broken into Chitin Oligosaccharide (COS) by chitinase [28]. COS shows diverse pharmacological potential. COS has functions against cancer and inflammation [29] and is a potential anti-inflammatory drug by activating tumor necrosis factor (TNF) alpha [30]. From the information, COS may be effective for controlling surgical infection. The main aim of the present work is to investigate the effects of COS on surgical infection and related molecular mechanisms in AIS patients. Meanwhile, antibiotics-caused side effects and the levels of related oxidant-related molecules and inflammatory cytokines were measured.

## 2. Results

### 2.1. The Bacteria Isolated from AIS Patients

The following bacteria were isolated from infected wounds of AIS patients and confirmed by 16s rRNA: *Pseudomonas aeruginosa*, *Burkholderia cepacia*, *Burkholderia cenocepacia*, *Acinetobacter baumannii*, *Acinetobacter lwoffii*, *Klebsiella pneumoniae*, *Escherichia coli*, *Staphylococcus aureus* and *Providencia stuartii*. These isolates represent the most frequently encountered resistance types. The resistance to aminoglycosides and fluoroquinolones is widely found in isolated *P. aeruginosa* [31]. *P. aeruginosa* can cause acute and chronic infections. New treatment strategies against *P. aeruginosa* infection are in especially high demand for its increasing resistance to the antibiotic Azithromycin. The overexpression of PA3297 (a DEAH-box helicase; DEAH, Asp-Glu-Ala-His) was found to be caused by the interaction between Azithromycin and ribosomes. The mutant PA3297 will increase unprocessed 23TS-5S rRNA in the presence of Azithromycin, which increases the sensitivity to Azithromycin-reduced effects, suggesting the bacterial reactions to counteract the effects of Azithromycin [32]. Lipopolysaccharides/lipooligosaccharides are abundant in the cell surface and increase antibiotics resistance in most Gram-negative bacteria. *A. baumannii* can develop resistance to Azithromycin via the loss of lipopolysaccharides (LPS) with mutant genes, which are associated with lipid synthesis [33]. Long-term therapy of Ceftazidime for surgical infection will increase beta-lactam resistance in Burkholderia and results in treatment failure [34]. *A. lwoffii* shows multi-drug resistance (MDR), which may be caused by a bla (NDM-1)-bearing plasmid, pNDM-BJ01, and mutant pNDM-BJ02 [35]. MDR is common in *E. coli* and its resistance to antibiotics has been reported to be associated with virulence [36]. The MDR of *S. aureus* has been found to be associated with class 1 and 2 integrons and gene cassettes [37].

### 2.2. Effects of COS on Isolated Bacteria

**Figure 1:**
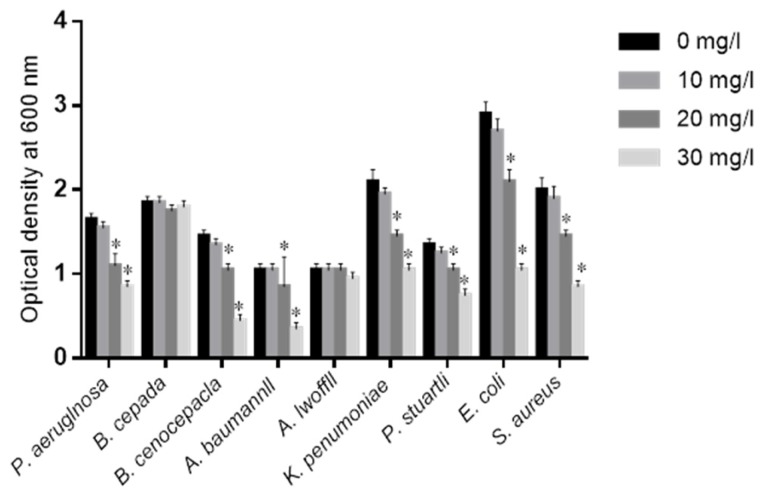
The effects of chitin oligosaccharide (COS) on the growth of clinically isolated pathogens. All pathogens were cultured for 24 h with different concentrations of COS at 37 °C. All data were presented as average value ± standard derivation (S.D.) * *p* < 0.05 vs. 0 mg/L COS. There is a statistical significance of differences if *p* < 0.05.

COS was proved to prevent MDR bacteria growth in a dose-dependent way after one-day culture (Figure 1). This effect was dose dependent for the concentration range investigated (0, 10, 20 and 30 mg/L). Generally, the growth rates of these bacteria were decreasing with increasing concentration of COS. The inhibitory functions were prominent for the pathogens *P. aeruginosa*, *B. cenocepacla*, *A. baumannii*, *K. pneumoniae*, *E. coli*, *S. aureus* and *P. stuartli*.

### 2.3. COS Reduces the Antibiotics Resistance of MDR Bacteria

The effects of COS on the dose of antibiotics for fighting against MDR bacteria were measured. The antibiotics (Azithromycin, Erythromycin, Cloxacillin, Aztreonam and Ceftazidime) were selected for detecting antibiotic-resistant pathogens (Table 1).

As Table 1 showed, COS addition decreased the minimum inhibitory concentration (MIC) of most antibiotics, including Azithromycin, Erythromycin, Cloxacillin, Aztreonam and Ceftazidime. Among these antibiotics, the MIC of *P. aeruginosa* was decreased from 19 to 0.5 μg/mL for Ceflazidime, suggesting the bacteria could be inhibited by reducing antibiotics resistance via COS. The MIC of *P. aeruginosa* was decreased from 250 to 6 μg/mL for erythromycin with the increase of COS dose from 0 to 30 mg/L. COS could reduce the antibiotics resistance of *B. cepacia* for Azithromycin, Erythromycin, Cloxacillin, Aztreonam and Ceftazidime from 30, 60, 120, 250 and 60 to 0.1, 9, 2, 2 and 0.5 μg/mL, respectively. The inhibitory functions of COS for antibiotic resistance were prominent for other pathogens except for *P. stuartli* by reducing the MICs of most antibiotics. COS had similar inhibitory functions for antibiotics resistance if combined antibiotics were used (data were not shown). A lesser effect was observed with Gram-positive isolates.

### 2.4. The Measurement of Resistance to COS

As Table 2 showed, MIC analysis indicated that *P. aeruginosa* had no resistance to COS although the concentration of COS was increased and the bacteria were cultured for three months in the media with COS. There was no statistical significance of differences among the groups with different concentrations of COS, suggesting no resistance to COS in the pathogens. Similarly, the increasing concentration of COS could not increase the drug resistance of all other pathogens (*p* > 0.05).

### 2.5. Baseline Characters

A total of 312 AIS subjects were selected in the present study and the average follow-up period was one month (which ranged from two to ten weeks). Seventy-one (31.4%) patients (group B) received antimicrobials until drain removal (range 3–5 days). There was no statistical significance of differences among three groups, including average age, gender, body mass index (BMI), lifestyles and the symptoms of AIS (scoliosis curve type, mean number of levels fused per patient, intra-operative transfusion, post-operative transfusion and duration of drain left in situ). There was no statistical significance of differences in all parameters found among three groups (*p* > 0.05, Table 3). To avoid instrument interference for surgery, different instruments were also compared among three groups. No statistical significance of differences was found either (Table 4, *p* > 0.05). Different surgical levels would cause different results, and thus the fused levels, surgical duration, blood loss and spine drains were also compared here. The results showed that there was no statistical significance of differences in these factors among three groups (Table 5, *p* > 0.05).

### 2.6. COS Were Potential Adjuvants of Antibiotics for Preventing Surgical Infection

All patients were randomly and evenly assigned into three groups according to different therapies after operation: control group (CG, the patients received one-gram alternative drugs Azithromycin/Erythromycin/Cloxacillin/Aztreonam/Ceftazidime or combined in one gram daily), experiment group (EG, the patients received 20 mg COS and half-dose antibiotics daily) and placebo group (PG, the patients received 20 mg placebo and half-dose antibiotics daily). The overall rates of SSI were 1%, 2% and 10% for spine wounds and 0%, 1% and 3% for the iliac crest wounds in CG, EG and PG, respectively. The patients had lower SSI rates for spine wounds in CG and EG when compared with PG (Table 6, *p* < 0.05). Although the patients also had lower SSI rates for iliac wounds among three groups, there was no statistical significance of differences (Table 6, *p* > 0.05). The results may be caused by a small population in the present work and result in a limited number of the patients with iliac wound. The present findings indicated that COS were potential adjuvants in antibiotics therapy for preventing surgical infection since only half-dose antibiotics were used in EG.

### 2.7. COS Prevented the Side Effects Caused by Antibiotics

Although most antibiotics can control most surgical infection well, the adverse effects are obvious, which limit their clinical use. Just as we proposed, the side effects were higher in CG than in EG and or PG (Table 7, *p* < 0.05). Fewer side effects were found in EG and PG groups. Most side effects included gastric upset, nausea, headache, vomiting, diarrhea, abdominal pain, seizure, chills, malaise, anxiety and fever in CG.

### 2.8. COS Improves the Biochemical Parameters of AIS Patients

Before the experiment, serum biochemical index analysis showed that there was no statistical significance of differences for serum levels of SOD (superoxide dismutase), GSH (reduced glutathione), ALT (alanine aminotransferase) and AST (aspartate amino-transaminase) among three groups (Table 8) (*p* > 0.05). Comparatively, serum biochemical index analysis showed that serum ALT and AST, reached the highest level in PG when compared with the other two groups after an average of one-month follow-up (Table 8) (*p* < 0.05). In contrast, the serum SOD and GSH reached the lowest level in PG as compared to two other groups (Table 8) (*p* < 0.05). COS increased the levels of SOD and GSH, and reduced the serum levels of ALT and AST.

### 2.9. COS Reduced Relative mRNA Levels of Inflammatory Cytokines (IL-6 and TNF Alpha)

**Figure 2:**
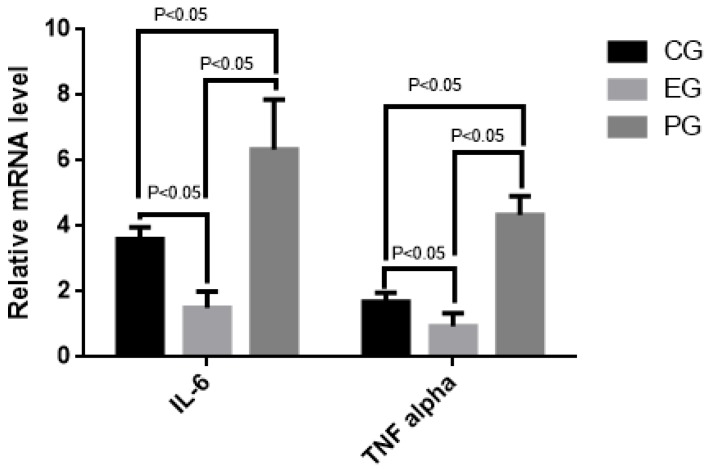
Real-time qRT-PCR analysis of the effects of COS on relative mRNA levels of interleukin (IL-6) and tumor necrosis factor (TNF) alpha. All data were presented as average value ± S.D. There is statistical significance of differences if *p* < 0.05.

qRT-PCR analysis indicated that the mRNA levels of IL-6 and TNF alpha were higher in PG than in CG, and EG (Figure 2) (*p* < 0.05). Furthermore, the mRNA levels of IL-6 and TNF alpha were higher in CG than in EG (Figure 1) (*p* < 0.05). The results suggest that COS reduces the mRNA levels of IL-6 and TNF alpha.

### 2.10. COS Reduced Relative Protein Levels of Inflammatory Cytokines (IL-6 and TNF Alpha)

**Figure 3:**
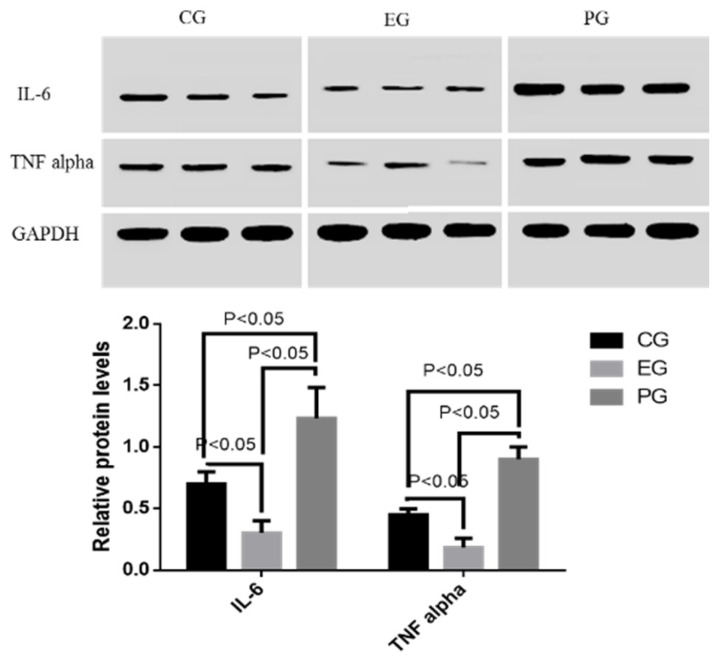
Western blot analysis of the effects of COS on relative protein levels of IL-6 and TNF alpha. All data were presented as average value ± S.D. There is statistical significance of differences if *p* < 0.05.

Just as qRT-PCR analysis, Western blot results indicated that the protein levels of IL-6 and TNF alpha were higher in PG than in CG, and EG (Figure 3) (*p* < 0.05). Furthermore, the protein levels of IL-6 and TNF alpha were higher in CG than in EG (Figure 3) (*p* < 0.05). The results suggest that COS reduces the protein levels of IL-6 and TNF alpha.

## 3. Discussion

Present results showed that COS reduced the dose and side effects of antibiotics, and SSI rates, which may be associated with antioxidant and anti-inflammation activities of COS. Infection will result in the increase of reactive oxygen species (ROS) [38] by affecting ROS-related molecules SOD [39], GSH [40], ALT [41] and AST [42]. COS improved antioxidant activities by increasing the activities of the anti-oxidant enzymes SOD and GSH, and decreasing the levels of oxidative-stress-related biomarkers ALT and AST (Table 8, *p* < 0.05). IL-6 and TNF alpha are involved with the infection inflammation and their levels will be increased. For instance, IL-6 trans-signaling plays an important role in the angiogenesis of the peritoneal membrane [43], in which IL-6 is an important inflammatory cytokine. Vascular inflammation is an important reason for causing atherosclerosis. High-level TNF alpha will induce vascular inflammation. TNF alpha neutralizing antibodies have been administered to treat many inflammatory disorders [44]. COS may improve anti-inflammatory activity by reducing levels of IL-6 and TNF alpha (Figure 2 and Figure 3, *p* < 0.05). All the results may contribute to the reduction of SSI rates after COS treatment (Table 6, *p* < 0.05).

Other reports showed that COS performed its anti-inflammatory functions via activating nuclear factor-kappa B [45], cyclooxygenase-2 [46], and inducible nitric oxide synthase [45]. Chitin is essential structural polysaccharide of many fungal pathogens and plays an important role in human immune responses. COS inhibited LPS-induced inflammation and contributed to human immune response when the pathogens were killed. Furthermore, NOD2 and TLR9 were regarded as chitin receptors and regulated inflammatory conditions by preventing the expression of chitinases, whose activity was critical to inflammatory conditions. COS has an important role in infectious and allergic disorders [47]. However, chitin has no such effects because it is hard to be dissolved in solution and must be digested into COS by chitinase. In this way, COS can be absorbed well by humans.

Antibiotics have been widely used in the therapy of surgical infection. However, the development of MDR bacteria is a big challenge for an anti-infection therapy. COS showed potential activities against MDR microorganisms. Present work proved that long-term use of COS decreased the MIC values of most MDR bacteria, which were isolated clinically. The reduction of MIC of most antibiotics will be beneficial to effectively control surgical infection. On the other hand, COS had been demonstrated to be effective against various antibiotics-resistant bacteria. COS provided a new way to make use of present antibiotics in low dosage. COS promoted therapeutic progression in treating surgical infection, which was caused by *Burkholderia* and *Acinetobacter* species. These pathogens are aggressively treated and often develop antibiotics resistance after long-term use of antibiotics.

Our subsequent work proved that COS was safe and tolerable in AIS patients. COS can be widely used in food and medical fields for its lesser side effects and safety for human health. Present work also proved that long-term use of high-concentrations of COS will not increase the resistance of most pathogens for COS. Although chitin cannot be used well, it cannot be solved in solution. COS can be produced with high purification via chitinase. The method is superior to the normal chemical method, which can cause environmental contamination.

There were some limitations for present work: (1) the population was still in a small sample size when different antibiotics and pathogens were considered; (2) we did not explore the molecular mechanisms for the development of antibiotics resistance of the clinically isolated pathogens; (3) AIS is a very complex disorder and many other affected factors may be not considered in the present work; (4) the anti-inflammatory and antioxidant mechanisms of COS remained widely unknown and could not be used alone as an experimental group; (5) present findings showed that fewer side effects were found in EG and PG groups compared to CG. Most of the side effects included gastric upset, nausea, headache, vomiting, diarrhea, abdominal pain, seizure, chills, malaise, anxiety and fever in CG. Half-dose antibiotics were used in EG and PG groups, and thus side effects would be reduced since they were caused by these antibiotics; and (6) the bacterial culture experiments showed that COS decreased MIC values for most of the bacteria. All of these pathogens were clinically isolated from human subjects. However, the effects of COS on these bacteria inside human subjects were not performed. Further work is still needed in the future.

## 4. Materials and Methods

### 4.1. Materials

COS was purchased from Qingdao BZ-Oligo Co., Ltd. (Qingdao, China). The COS was made from crab shells and obtained by chitinase digestion, chemical derivatization and column chromatography. Briefly, chitins were treated with alkali to increase solubility of the substrates. Chitinase was expressed in *Pichia pastoris* and purified [48]. In addition, 1 mL (1 mg/mL) of purified chitinase was added to one liter of chitin hydrolysis (1% chitin, *w*/*v*). The digesting reaction was performed at 39 °C and pH 5 for half an hour. The digesting solution was ultrafiltrated with a membrane NMWL of 3 kDa (Millipore Corporation, Billerica, MA, USA). Filtrated solution was further purified via gel filtration chromatography (Amersham Pharmacia Biotech Inc., Piscataway, NJ, USA). The COS with the degrees of polymerization (DP) ranging from 2 to 10 was obtained, and followed by spray drying.

### 4.2. Participants

Before the experiments, all protocols were approved by the ethical committee of our hospital. From 3 March 2014 to 16 May 2015, a total of 312 AIS patients who had undergone posterior spinal fusion were collected.

### 4.3. Inclusion Criteria

All the patients meet the diagnostic criteria and classification criteria of AIS [49]; and the ages ranged from 10 to 16 years old. For the patients in the coronal range of 25° to 45°, they require surgical treatment.

### 4.4. Exclusion Criteria

Those who do not meet the diagnostic criteria; had experienced surgery more than twice; patients did not meet the inclusion age; coronal plane angle: angle jump angle less than 25°; patients wo had received other relevant treatment, which may affect the effect of present experimental results; patients had other diseases, including cardiovascular system, liver, kidney, hematopoietic system, endocrine system diseases and cancer; patients had allergic disorders; patients had brain diseases; and patients who did not sign the informed consent.

### 4.5. Patients Grouping

All patients were randomly and evenly assigned into three groups according to different therapies as above mentioned: CG, EG and PG groups. The average follow-up was one month (from two weeks to ten weeks), and infection severity and side effects were analyzed. Analysis was performed to evaluate differences in post-operative variables among three groups.

### 4.6. Baseline Measurement

The baseline characters (age, gender, lifestyle, infection rate and daily calorie uptake) were compared among three groups. The following parameters (related with surgical risks) were also considered: blood loss in the surgery, surgical length, vertebral levels fused and anchor points, post-operative drain collection and its duration were recorded. For patients who received antimicrobials until drain removal, the number of days that the drug was administered was recorded. Serious adverse events were recorded including gastric upset, nausea, headache, vomiting, diarrhea, abdominal pain, seizure, chills, malaise, anxiety and fever.

### 4.7. Measurement of Surgical Infection

Presently, there is no uniform standard for the diagnosis of orthopedic infection. Postoperative infection of orthopedic surgery can be determined if the patients had purulent secretion exudation, clinical or surgical or pathological or imaging diagnosis of deep incision. There are abscesses, sinus secretions, joint punctures, and intraoperative lesions in the fluid culture that can be diagnosed as pathogens and other infections. In addition, white blood cell count (WBC), neutrophils, C-reactive protein (CRP), erythrocyte sedimentation rate (ESR), body temperature and other clinical abnormalities can also help to diagnose postoperative infection. Increased WBC is often considered as an indicator of the diagnosis of orthopedic infection. Postoperative infection was measured with clinical infection, such as skin, intravenous tissue, or muscles over the fascial layer. For each patient with SSI, the organism was isolated, and its antimicrobial sensitivity and subsequent management were recorded. The species of infected bacteria were identified by 16S rRNA.

### 4.8. Measurement of Anti-Bacteria Activities of COS

To explore the inhibitory functions of COS on isolated pathogens, the effects of COS on these pathogens were analyzed according to an earlier report [50]. The effects of COS on bacterial growth were measured by using different concentrations of COS (from 0 to 30 mg/L). All bacteria were cultured in tryptone soya broth for 20 h and transferred into a new 50 mL tube with 5 mL tryptone soya broth with different concentrations (from 0 to 30 mg/L) and cultured at 37 °C for 24 h. Cellular concentrations were measured at OD_600nm_ (1 OD = 10^7^ cells/mL). Anti-bacterial functions of COS were further determined by using MIC [51]. A single colony was cultured for 20 h in tryptone soya broth and diluted in Phosphate salt buffer until the OD_600nm_ was 0.01 (10^7^ CFU/mL). Serial-diluted antibiotics were added to the above broth or the broth with different concentrations of COS in a 96-well plate and cultured at 37 °C for 20 h. MICs were calculated as the lowest concentration when no growth was observed.

### 4.9. Measurement for the Resistance to COS after Long-Term Culture

All isolated pathogens were sub-cultured (one time within 24 h) in tryptone soya broth with different concentrations of COS (from 0 to 30 mg/L) for three months. MIC was analyzed on every tenth day to find if there was resistance of pathogens to COS with different concentrations of COS.

### 4.10. Biochemical Analysis

Oxidative stress was analyzed because oxidative stress is an important risk factor for the development of AIS. The serum activity of SOD was measured by the formazan-WST (water-soluble tetrazolium salt) method [52]. The serum concentration of GSH was determined by using Dithiobis-2-nitrobenzoic acid (DTNB) [53]. The serum concentrations of AST and ALT were evaluated by using the Hitachi 7170A/7180 Biochemical Analyzer (Hitachi, Japan). Serum levels of IL-6 and TNF alpha were measured by using Human IL-6 Quantikine ELISA Kit D6050 and Human TNF-alpha Quantikine ELISA Kit DTA00C ELISA kits (R&D Systems Inc., Minneapolis, MN, USA), respectively.

### 4.11. qRT-PCR

Total hepatic RNA was exacted and purified by using a RNA purification kit. cDNA was produced based on the instructions of the RT-PCR kit. qRT-PCR was performed to assay the mRNA levels of IL-6 (Forward primer, 5’-cacaacagaccagtatatac-3’; Reverse primer, 5’-gtatttctggaagtttcag-3’) and TNF alpha (Forward primer, 5’-gtggcgggggccaccacgctc-3’; Reverse primer, 5’-cgagttttgagaagatgatc-3’) genes. GAPDH (Glyceraldehyde 3-phosphate dehydrogenase) was used as an internal control to standardize the copy number (*C*t value) of each sample. qRT-PCR was performed on the CFX96 Touch Real-Time PCR Detection System (Bio-Rad, Hercules, CA, USA). The mean *C*t value represented the mRNA levels of individual genes.

### 4.12. Western Blot Analysis

Protein was extracted and the concentration was determined by Bradford protein assay kit (Beyotime Biotechnology, Beijing, China). In addition, 30-µg proteins were taken from each group, separated by 12% SDS-PAGE, and then transferred to a polyvinylidene difluoride (PVDF) membranes (Millipore Corporation, Bedford, MA, USA), which was blocked by 5% non-fat milk. The membrane was treated with primary antibodies at 4 °C for 10 h. Secondary antibodies were added and incubated for one hour. Protein bands were shown after one-hour exposure with GE's Amersham ECL+ Chemiluminescent CCD camera (City, US State abbrev. if applicable, Country). The protein level was indicated as the value according to the relative ratio to control GAPDH.

### 4.13. Statistical Analysis

All data were presented as mean values ± S.D. Analysis of variance (ANOVA) test was used to compare the changes in post-operative infection rate among three groups. Chi-square tests and student’s *t*-tests were used for analyzing the difference between two groups. Statistical analysis was carried out by using SPSS 20.0 (SPSS Inc., Chicago, IL, USA). There was statistical significance of differences if *p* was <0.05.

## 5. Conclusions

COS reduced clinical pathogens resistantance to normal antibiotics and improved the management of antibiotics. COS can reduce the dose of antibiotics and control the risk of the development of antibiotics resistance in some clinical pathogens. The exact COS concentration and clinical pathogens still need to be studied in the future. COS should be developed as a kind of potential adjuvant of antibiotics therapy for clinical infection.

## Figures and Tables

**Table 1:** MICs of antibiotics alone and with increasing concentrations (0, 10, 20 and 30 mg/L) of COS for a range of antibiotics-resistant clinical isolates (Antibiotic MIC, mg/L).

Antibiotics	COS	PA	BC	BC1	AB	AL	KP	EC	SA	PS
Azithromycin	0	7	30	3	17	0.2	500	8	0.1	8
	10	3	16	2	4	0.1	250	4	0.1	2
	20	2	5	1	0.5	0.1	60	1	0.1	1
	30	0.5	0.1	0.5	0.02	0.1	32	0.5	0.1	0.1
Erythromycin	0	250	60	68	9	0.2	250	30	0.1	20
	10	126	48	38	4	0.1	120	18	0.1	16
	20	30	17	16	1	0.1	65	8	0.1	0.1
	30	6	9	8	0.3	0.1	20	4	0.1	0.1
Cloxacillin	0	1000	120	39	2	0.1	2	4	0.1	0.1
	10	1000	60	16	2	0.1	2	4	0.1	0.1
	20	1000	17	8	1	0.1	1	4	0.1	0.1
	30	1000	2	2	1	0.1	1	2	0.1	0.1
Aztreonam	0	34	250	129	678	30	4000	1000	512	0.1
	10	14	100	32	312	15	2000	510	234	0.1
	20	9	8	3	250	4	500	250	249	0.1
	30	3	2	1	126	1	250	64	244	0.1
Ceftazidime	0	19	60	8	778	2	4230	4123	12	8
	10	8	8	4	546	2	2078	3124	8	4
	20	1	2	1	312	0.5	1536	2341	2	2
	30	0.5	0.25	0.25	156	0.1	1324	1097	1	1

Note: MIC, minimal inhibitory concentration; COS, chitin oligosaccharide; PA, *P. aeruginosa*; BC, *B. cepacia*; BC1, *B. cenocepacla*; AB, *A. baumannii*; AL, *A. lwoffii*; KP, *K. pneumoniae*; EC, *E. coli*; SA, *S. aureus*; PS, *P. stuartli*. A maximum dose for an adult is 10 mg/kg for Azithromycin, 50 mg/kg for erythromycin, 100 mg/kg for Cloxacillin, 200 mg/kg for Aztreonam, and 150 mg/kg for Ceftazidime daily.

**Table 2:** MICs of antibiotics for clinical isolates with increasing concentrations of COS (mg/L).

COS	PA	BC	BC1	AB	AL	KP	EC	SA	PS
0	10 ± 1.2	5 ± 0.6	8 ± 1	20 ± 2.6	4 ± 0.6	106 ± 11	9 ± 1.1	2 ± 0.1	11 ± 1.3
10	12 ± 1.5	5 ± 0.9	9 ± 1.1	18 ± 2.2	4 ± 0.8	100 ± 10	11 ± 0.8	2 ± 0.2	9 ± 1.6
20	11 ± 1.4	6 ± 0.7	10 ± 1.2	17 ± 2.4	3 ± 0.4	95 ± 9	12 ± 0.9	3 ± 0.2	12 ± 1.5
30	12 ± 1.3	5.5 ± 0.6	10 ± 1.3	21 ± 1.6	5 ± 0.6	112 ± 12	14 ± 1.5	4 ± 0.4	13 ± 1.1
*p* values	0.56	0.73	0.4	0.23	0.18	0.09	0.15	0.10	0.27

Note: PA, *P. aeruginosa*; BC, *B. cepacia*; BC1, *B. cenocepacla*; AB, *A. baumannii*; AL, *A. lwoffii*; KP, *K. pneumoniae*; EC, *E. coli*; SA, *S. aureus*; PS, *P. stuartli*. There is statistical significance of differences if *p* < 0.05.

**Table 3:** Baseline characters (*n* = 104 for each group).

Parameters	CG, Mean (SD; Range)	EG, Mean (SD; Range)	PG, Mean (SD; Range)	*p*
Age, years	12.03 (SD 2.64; 10–16)	11.80 (SD 2.28; 10–16)	12.37 (SD 2.70; 11–26)	0.86
Gender, male/female	74/30	76/28	70/34	0.65
BMI	18.7 (SD 2.8; 13.42–28.27)		18.93 (SD 3.62; 12.29–30.49)	0.28
Smoking	23	20	18	0.65
Scoliosis curve type (MT/DM/DT/TM/TL)	58/22/19/0/5	55/25/17/2/5	59/21/16/4/4	0.26
Mean number of levels fused per patient	9.26 (SD 2.06; 7–13)	9.38 (SD 2.21; 7–13)	9.57 (SD 2.23; 7–13)	0.78
Surgical duration (minutes)	253.88 (SD 62.15; 198–487)	287.95 (SD 61.85; 178–483)	279.94 (SD 76.7; 196–437)	0.53
Mean number of anchor points per patient	10.1 (SD 2.49; 67–20)	11.1 (SD 2.69; 7–21)	9.2(SD 2.06; 7–18)	0.39
Post-operative transfusion, mL	Blood: 198.26 (SD 149.80; 0–745) FFP: 21.32 (SD 77.23; 0–545)	Blood: 218.26 (SD 169.80; 0–721) FFP: 20.42 (SD 76.63; 0–521)	Blood: 207.68 (SD 282.43; 0–986) FFP: 22.48 (SD 75.37; 0–530)	0.21
Post-operative spine drain, mL	Total drain: 218.01 (SD 221.76; 5–1500)	Total drain: 473.02 (SD 355.40; 5–1320)	Total drain: 463.01 (SD 345.90; 5–1640)	0.41
Post-operative duration of drain in situ, days	3.2 (SD 0.68; 2–7)	3.0 (SD 0.61; 2–7)	3.3 (SD 0.75; 2–7)	0.36

Note: All patients were randomly and evenly assigned into three groups according to different therapies after operation: control group (CG, the patients received one-gram alternative drugs Azithromycin/Erythromycin/Cloxacillin/Aztreonam/Ceftazidime or combined in one gram daily), experiment group (EG, the patients received 20 mg COS and half-dose antibiotics daily) and placebo group (PG, the patients received 20 mg placebo and half-dose antibiotics daily). SD, standard deviation. ANOVA test and chi-square test were performed for comparing the statistical significance of difference among three groups. There is statistical significance of differences if *p* < 0.05.

**Table 4:** Comparison of instruments among three groups.

Instrument	CG	EG	PG	*p*-Value
All pedicle screw constructs	35	30	34	0.73
Pedicle screws and hook constructs	42	48	46	0.69
All hook constructs	10	10	10	1.00
Pedicle screws, hooks and sublaminar wire construct	11	10	8	0.77
Pedicle screw and sublaminar wire construct	6	6	6	1.00

Note: chi-square test was performed for comparing the statistical significance of difference among three groups. There is statistical significance of differences if *p* < 0.05.

**Table 5:** Surgical operation.

Levels Fusion		T6-L2	T2-L1	T2-L2	T5-L4	T7-L2	T6-12	T4-11
Numbers	CG	35	24	12	9	10	8	6
	EG	33	26	13	8	11	7	6
	PG	34	25	14	7	9	8	7
*p*-value		1						
Surgical duration (minutes)	CG	250 ± 45	263 ± 51	298 ± 46	305 ± 62	527 ± 71	296 ± 43	248 ± 32
	EG	242 ± 49	251 ± 59	277 ± 53	311 ± 53	512 ± 63	280 ± 37	239 ± 28
	PG	256 ± 40	266 ± 52	302 ± 43	299 ± 47	531 ± 58	301 ± 26	254 ± 21
*p*-value		0.91	0.85	0.69	0.27	0.53	0.44	0.75
Blood loss (mL)	CG	537 ± 214	587 ± 196	716 ± 243	436 ± 178	NA	NA	NA
	EG	498 ± 253	502 ± 245	642 ± 215	451 ± 210	NA	NA	NA
	PG	399 ± 128	546 ± 237	597 ± 204	407 ± 185	NA	NA	NA
*p*-value		0.12	0.26	0.37	0.55			
Spine drain (mL)	CG	270 ± 123	346 ± 188	164 ± 49	105 ± 31	541 ± 103	90 ± 21	100 ± 17
	EG	244 ± 105	320 ± 171	154 ± 33	96 ± 18	501 ± 83	84 ± 13	93 ± 11
	PG	252 ± 98	305 ± 124	137 ± 41	99 ± 27	527 ± 66	89 ± 17	102 ± 22
*p*-value		0.18	0.29	0.08	0.57	0.62	0.71	0.41

Note: chi-square test was performed for comparing the statistical significance of difference among three groups. There is statistical significance of differences if *p* < 0.05.

**Table 6:** The rates of surgical site infections.

Wound Category	CG	EG	PG	P1	P2	P3
Spine wound	1	2	10	1	0.004	0.005
Iliac wound	0	1	3	1	0.245	0.607

Note: chi-square test was performed for comparing the statistical significance of difference for the rates of surgical site infections among three groups. The infection was not considered if the infection duration was less than three days. The infection would be counted if the duration was more than three days. P1, CG vs. EG; P2, CG vs. PG; P3, EG vs. PG. There is statistical significance of differences if *p* < 0.05.

**Table 7:** Side effects of AIS patients in different groups.

Side Effects	CG	EG	PG	P1	P2	P3
gastric upset	15	3	1	0.003	0.000	0.614
nausea	13	1	0	0.001	0.000	1.000
headache	15	3	1	0.003	0.000	0.614
vomiting	20	4	2	0.000	0.000	0.679
diarrhea	7	0	0	0.210	0.210	1.000
abdominal pain	10	0	1	0.004	0.005	1.000
seizure	3	0	1	0.245	0.614	1.000
chills	6	1	1	0.124	0.124	1.000
malaise	5	1	1	0.214	0.214	1.000
anxiety	6	0	1	0.038	0.124	1.000
fever	8	0	1	0.012	0.041	1.000

Note: AIS, adolescent idiopathic scoliosis; chi-square test was performed for comparing the statistical significance of difference among three groups. P1, CG via EG; P2, CG via PG; P3, EG via PG. There is statistical significance of differences if *p* < 0.05.

**Table 8:** Biochemical parameters of enzyme activities in AIS patients.

Stages	Group (*n* = 10)	SOD (U/mL)	GSH (pg/mL)	ALT (pg/mL)	AST (pg/mL)
Before experiment	CG	26.24 ± 3.16	22.15 ± 2.04	44.12 ± 10.13	100.32 ± 25.67
	EG	24.25 ± 4.16	23.23 ± 1.93	47.79 ± 8.40	108.26 ± 19.64
	PG	25.34 ± 2.32	21.28 ± 1.74	43.79 ± 12.36	103.42 ± 24.48
	*p*-value	0.56	0.74	0.12	0.68
After experiment	CG	22.73 ± 3.28 Δ,●	21.09 ± 3.06 Δ	48.31 ± 8.25 Δ,●	118.36 ± 26.82 Δ,●
	EG	28.29 ± 5.13 *,●	25.44 ± 2.01 *,●	38.1 9± 6.25 *,●	76.34 ± 14.38 *,●
	PIG	15.32 ± 5.71 *,Δ	14.26 ± 4.39 *,Δ	53.69 ± 18.31 *,Δ	89.46 ± 27.52 *,Δ
	*p*-value	0.01	0.01	0.01	0.01

Note: AIS, adolescent idiopathic scoliosis; SOD, superoxidedismutase; GSH, glutathione; AST, aspartate transaminase; ALT, alanine transaminase. * *p* < 0.05 vs. the control group (CG); Δ *p* < 0.05 vs. the experimental group (EG); ● *p* < 0.01 vs. the placebo group (PG). There is statistical significance of differences if *p* < 0.05.
